# Population-Based Surveillance for Birth Defects Potentially Related to Zika Virus Infection — 22 States and Territories, January 2016–June 2017

**DOI:** 10.15585/mmwr.mm6903a3

**Published:** 2020-01-24

**Authors:** Ashley N. Smoots, Samantha M. Olson, Janet Cragan, Augustina Delaney, Nicole M. Roth, Shana Godfred-Cato, Abbey M. Jones, John F. Nahabedian, Jane Fornoff, Theresa Sandidge, Mahsa M. Yazdy, Cathleen Higgins, Richard S. Olney, Valorie Eckert, Allison Forkner, Deborah J. Fox, Amanda Stolz, Katherine Crawford, Sook Ja Cho, Mary Knapp, Muhammad Farooq Ahmed, Heather Lake-Burger, Amanda L. Elmore, Peter Langlois, Rebecca Breidenbach, Amy Nance, Lindsay Denson, Lisa Caton, Nina Forestieri, Kristin Bergman, Brian K. Humphries, Vinita Oberoi Leedom, Tri Tran, Julie Johnston, Miguel Valencia-Prado, Stephany Pérez-González, Paul A. Romitti, Carrie Fall, J. Michael Bryan, Jerusha Barton, William Arias, Kristen St. John, Sylvia Mann, Jonathan Kimura, Lucia Orantes, Brennan Martin, Leah de Wilde, Esther M. Ellis, Ziwei Song, Amanda Akosa, Caroline Goodroe, Sascha R. Ellington, Van T. Tong, Suzanne M. Gilboa, Cynthia A. Moore, Margaret A. Honein

**Affiliations:** ^1^Division of Birth Defects and Infant Disorders, National Center on Birth Defects and Developmental Disabilities, CDC; ^2^Illinois Department of Public Health; ^3^Massachusetts Department of Public Health; ^4^California Department of Public Health; ^5^Indiana State Department of Health; ^6^New York State Department of Health; ^7^Virginia Department of Health; ^8^Minnesota Department of Health; ^9^New Jersey Department of Health; ^10^Florida Department of Health; ^11^Texas Department of State Health Services; ^12^Utah Department of Health; ^13^Oklahoma State Department of Health; ^14^North Carolina Department of Health and Human Services; ^15^South Carolina Department of Health and Environmental Control; ^16^Louisiana Department of Health; ^17^Puerto Rico Department of Health;^18^University of Iowa, Iowa City; ^19^Georgia Department of Public Health; ^20^Rhode Island Department of Health; ^21^Hawaii Department of Health; ^22^Vermont Department of Health; ^23^U.S. Virgin Islands Department of Health; ^24^Division of Reproductive Health, National Center for Chronic Disease Prevention and Health Promotion, CDC.

Zika virus infection during pregnancy can cause congenital brain and eye abnormalities and is associated with neurodevelopmental abnormalities (*1*–*3*). In areas of the United States that experienced local Zika virus transmission, the prevalence of birth defects potentially related to Zika virus infection during pregnancy increased in the second half of 2016 compared with the first half (*4*). To update the previous report, CDC analyzed population-based surveillance data from 22 states and territories to estimate the prevalence of birth defects potentially related to Zika virus infection, regardless of laboratory evidence of or exposure to Zika virus, among pregnancies completed during January 1, 2016–June 30, 2017. Jurisdictions were categorized as those 1) with widespread local transmission of Zika virus; 2) with limited local transmission of Zika virus; and 3) without local transmission of Zika virus. Among 2,004,630 live births, 3,359 infants and fetuses with birth defects potentially related to Zika virus infection during pregnancy were identified (1.7 per 1,000 live births, 95% confidence interval [CI] = 1.6–1.7). In areas with widespread local Zika virus transmission, the prevalence of birth defects potentially related to Zika virus infection during pregnancy was significantly higher during the quarters comprising July 2016–March 2017 (July–September 2016 = 3.0; October–December 2016 = 4.0; and January–March 2017 = 5.6 per 1,000 live births) compared with the reference period (January–March 2016) (1.3 per 1,000). These findings suggest a fourfold increase (prevalence ratio [PR] = 4.1, 95% CI = 2.1–8.4) in birth defects potentially related to Zika virus in widespread local transmission areas during January–March 2017 compared with that during January–March 2016, with the highest prevalence (7.0 per 1,000 live births) in February 2017. Population-based birth defects surveillance is critical for identifying infants and fetuses with birth defects potentially related to Zika virus regardless of whether Zika virus testing was conducted, especially given the high prevalence of asymptomatic disease. These data can be used to inform follow-up care and services as well as strengthen surveillance.

State and territorial health departments, in collaboration with CDC, conducted population-based surveillance for birth defects potentially related to Zika virus infection during pregnancy.* As previously described (*4*), data from medical records were abstracted for live births and pregnancy losses with any potentially Zika-related birth defect. Clinical expert review of verbatim descriptions was used to confirm case inclusion, and cases were assigned to one of four mutually exclusive categories.^†^ Because the case definition for birth defects potentially related to Zika virus infection has been updated to exclude neural tube defects (NTDs) and other early brain malformations and consequences of central nervous system dysfunction (*5*), the prevalence of cases with 1) brain abnormalities and/or microcephaly and 2) eye abnormalities without mention of a brain abnormality are reported. Prevalence estimates for NTDs and other early brain malformations during the study period, compared with brain and eye abnormalities in areas with widespread local transmission, are presented to support the updated case definition.^§^ Prevalence was calculated using the number of monthly live births reported by each jurisdiction.

Jurisdictions included in this report submitted data to CDC for the entire period (January 2016–June 2017). Jurisdictions were aggregated by level of local transmission of Zika virus: 1) widespread local transmission of Zika virus (Puerto Rico and the U.S. Virgin Islands); 2) limited local transmission of Zika virus (southern Florida counties and Texas Public Health Region 11); and 3) without local transmission of Zika virus.^¶^

Prevalence estimates for birth defects per 1,000 live births were calculated by group for each quarter. A PR (compared with the reference period, January–March 2016) was calculated for each quarter. PRs and CIs were calculated using Poisson regression. SAS (version 9.4; SAS Institute) was used to conduct all analyses.

During January 1, 2016–June 30, 2017, among 2,004,630 live births, 3,359 infants and fetuses with a birth defect potentially related to Zika virus infection were delivered to residents of the 22 jurisdictions, including 2,813 (83.7%) with brain abnormalities and/or microcephaly and 546 (16.3%) with eye abnormalities without mention of a brain abnormality (overall prevalence = 1.7 per 1,000 live births; 95% CI = 1.6–1.7) (Table 1). During the reference period, in areas with widespread local Zika transmission, limited local transmission, and without local transmission, prevalences were 1.3, 2.2, and 1.7 per 1,000 live births, respectively (Table 2).

**TABLE 1:** Population-based counts and prevalence of infants and fetuses with birth defects potentially related to Zika virus infection during pregnancy — 22 U.S. jurisdictions,* January 1, 2016–June 30, 2017

Characteristic	Brain abnormalities and/or microcephaly^†^ (n = 2,813 [83.7%])	Eye abnormalities without brain abnormalities^§^ (n = 546 [16.3%])	Total (N = 3,359 [100%])
**Prevalence^¶^ (95% CI)**	1.4 (1.4–1.5)	0.3 (0.3–0.3)	**1.7 (1.6–1.7)**
**Eye abnormalities, no. (%)**	289 (10.3)	—	**835 (24.9)**
Pregnancy outcome**			
Live birth, no. (%)	2,667 (95.7)	537 (99.3)	**3,204 (96.3)**
Neonatal death (≤28 days), no. (% of live births)	138 (5.2)	9 (1.7)	**147 (4.6)**
Pregnancy loss,^††^ no. (%)	119 (4.3)	4 (0.7)	**123 (3.7)**
**Zika virus laboratory testing for mothers or infants**			
Positive, no. (%)	64 (2.3)	9 (1.6)	**73 (2.2)**
Negative, no. (%)	103 (3.7)	15 (2.7)	**118 (3.5)**
No laboratory testing performed/NA,^§§^ no. (%)	2,646 (94.1)	522 (95.6)	**3,168 (94.3)**

**Abbreviations:** CI = confidence interval; NA = not applicable.

* 22 U.S. jurisdictions included births that occurred in California (selected counties), Florida (selected southern counties), Georgia (selected metropolitan Atlanta counties), Hawaii, Illinois, Indiana, Iowa, Louisiana, Massachusetts, Minnesota, New Jersey, New York (excluding New York City residents), North Carolina (selected regions), Oklahoma, Puerto Rico, Rhode Island, South Carolina, Texas (Public Health Regions 10, 11), the U.S. Virgin Islands, Utah, Vermont, and Virginia. Total live births = 2,004,630.

^†^ Congenital microcephaly (head circumference <3rd percentile for gestational age and sex and documentation of microcephaly or a small head in the medical record), intracranial calcifications, cerebral atrophy, abnormal cortical gyral patterns (e.g., polymicrogyria, lissencephaly, pachygyria, schizencephaly, gray matter heterotopia), corpus callosum abnormalities, cerebellar abnormalities, porencephaly, hydranencephaly, ventriculomegaly/hydrocephaly (excluding “mild” ventriculomegaly without other brain abnormalities), fetal brain disruption sequence (collapsed skull, overlapping sutures, prominent occipital bone, scalp rugae), and other major brain abnormalities.

^§^ Microphthalmia/anophthalmia, coloboma, cataract, intraocular calcifications, and chorioretinal anomalies (e.g., atrophy and scarring, gross pigmentary changes, excluding retinopathy of prematurity); optic nerve atrophy, pallor, and other optic nerve abnormalities.

^¶^ Per 1,000 live births.

** Thirty-two unknown pregnancy outcomes not included.

^††^ Included miscarriages, fetal deaths, and terminations. Not all programs reported pregnancy losses.

^§§^ Included cases where no testing was performed or testing status was unknown.

**TABLE 2:** Prevalence of birth defects potentially related to Zika virus infection* during pregnancy, by level of local transmission of Zika virus and quarter — 22 U.S. jurisdictions, January 1, 2016–June 30, 2017

Characteristic	Areas with widespread local transmission^†^ (n = 129 [3.8%])		Areas with limited local transmission^§^ (n = 340 [10.1%])		Areas without local transmission^¶^ (n = 2,890 [86.0%])	
	Prevalence**	PR^††^ (95% CI)	Prevalence**	PR^††^ (95% CI)	Prevalence**	PR^††^ (95% CI)
**Quarter**						
Jan–Mar 2016	1.3	Reference	2.2	Reference	1.7	Reference
Apr–Jun 2016	2.5	1.9 (0.9–4.0)	2.0	0.9 (0.6–1.3)	1.7	1.0 (0.9–1.1)
Jul–Sep 2016	3.0	2.3 (1.1–4.8)	2.0	0.9 (0.6–1.3)	1.7	1.0 (0.9–1.1)
Oct–Dec 2016	4.0	3.0 (1.4–6.1)	2.7	1.2 (0.9–1.7)	1.5	0.9 (0.8–1.0)
Jan–Mar 2017	5.6	4.1 (2.1–8.4)	1.9	0.8 (0.6–1.2)	1.5	0.9 (0.8–1.0)
Apr–Jun 2017	2.0	1.5 (0.7–3.5)	2.1	1.0 (0.7–1.4)	1.5	0.9 (0.8–1.0)
**Zika virus laboratory testing for mothers or infants**						
Positive, no. (%)	50 (38.8%)		7 (2.1%)		16 (0.6%)	
Negative, no. (%)	55 (42.6%)		27 (7.9%)		36 (1.3%)	
No laboratory testing performed/ NA,^§§^ no. (%)	24 (18.6%)		306 (90.0%)		2,838 (98.2%)	

**Abbreviations:** CI = confidence interval; NA = not applicable; PR = prevalence ratio.

* Fetuses and infants included those with 1) brain abnormalities and/or microcephaly or 2) eye abnormalities without mention of a brain abnormality included in the brain abnormalities and/or microcephaly category.

^†^ Jurisdictions with widespread local transmission of Zika virus during 2016–2017 included Puerto Rico and the U.S. Virgin Islands. Total live births for areas with widespread local transmission = 42,358.

^§^ Jurisdictions with limited local transmission of Zika virus during 2016–2017 included southern Florida counties and Texas Public Health Region 11. Total live births for areas with limited local transmission = 156,613.

^¶^ Jurisdictions without local transmission of Zika virus during 2016–2017 included California (selected counties), Georgia (selected metropolitan Atlanta counties), Hawaii, Illinois, Indiana, Iowa, Louisiana, Massachusetts, Minnesota, New Jersey, New York (excluding New York City residents), North Carolina (selected regions), Oklahoma, Rhode Island, South Carolina, Texas Public Health Region 10, Utah, Vermont, and Virginia. Total live births for areas without local transmission = 1,805,659.

** Per 1,000 live births.

^††^ Compared with reference, January–March 2016.

^§§^ Included cases where no testing was performed or testing status was unknown.

The prevalence of birth defects potentially related to Zika virus infection in widespread local transmission areas was significantly higher in three periods during July 2016–March 2017 compared with that during the reference period. Prevalence increased fourfold (PR = 4.1, 95% CI = 2.1–8.4) during January–March 2017 (5.6 per 1,000 live births), compared with that during the reference period (1.3 per 1,000) (Table 2), reaching a peak prevalence of 7.0 per 1,000 live births in February 2017 (Figure). In areas with limited local transmission, there was a 20% (PR = 1.2, 95% CI = 0.9–1.7) increase during October–December 2016 (2.7 per 1,000 live births) compared with that during the reference period (2.2 per 1,000), although the increase was not significant (Table 2). In areas without local transmission, there was also no significant difference in the prevalence of birth defects potentially related to Zika virus infection between the reference period and any of the subsequent quarters (Table 2). In widespread local Zika virus transmission areas, the significant prevalence increase was limited to brain abnormalities and/or microcephaly and eye abnormalities without mention of a brain abnormality; the prevalence of NTDs and other early brain malformations remained flat during the study period (Supplementary Figure, https://stacks.cdc.gov/view/cdc/84198).

**FIGURE:**
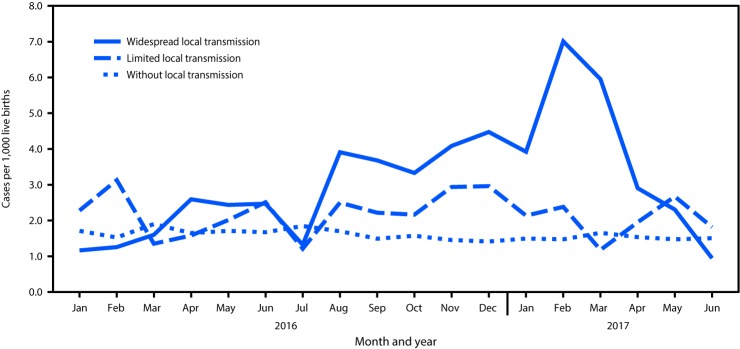
Prevalence of birth defects potentially related to Zika virus infection during pregnancy,* by level of local Zika virus transmission and month — 22 U.S. jurisdictions, January 2016–June 2017^†^^,^^§^^,^^¶^ * Fetuses and infants included those with 1) brain abnormalities and/or microcephaly or 2) eye abnormalities without mention of a brain abnormality included in brain abnormalities and/or microcephaly category. ^†^ Jurisdictions with widespread local transmission of Zika virus during 2016–2017 included Puerto Rico and the U.S. Virgin Islands. ^§^ Jurisdictions with limited local transmission of Zika virus during 2016–2017 included southern Florida counties and Texas Public Health Region 11. ^¶^ Jurisdictions without local transmission of Zika virus during 2016–2017 included California (selected counties), Georgia (selected metropolitan Atlanta counties), Hawaii, Illinois, Indiana, Iowa, Louisiana, Massachusetts, Minnesota, New Jersey, New York (excluding New York City residents), North Carolina (selected regions), Oklahoma, Rhode Island, South Carolina, Texas Public Health Region 10, Utah, Vermont, and Virginia.

Overall, most cases (3,168 [94.3%]) had no reported laboratory testing of maternal, placental, fetal, or infant specimens. Among the remaining 191 cases, laboratory evidence of confirmed or possible Zika virus infection was reported in at least one specimen for 73 (2.2%) cases, and 118 (3.5%) had negative Zika virus laboratory testing. In widespread local transmission areas, laboratory testing at any time in at least one specimen was reported for 105 of 129 (81.4%) cases; among the 105 cases with laboratory testing, 50 (47.6%) had laboratory evidence of confirmed or possible Zika virus infection.

## Discussion

The peak occurrence of birth defects potentially related to Zika virus infection in areas with widespread local transmission occurred in February 2017, 6 months after the reported peak of the Zika virus outbreak in these areas in August 2016 (*6*). This is consistent with other findings regarding the time between the peak of a Zika virus outbreak and recognition of an increase in potentially Zika-related birth defects (*7*). Approximately one half (47.6%) of cases with laboratory test results available in areas with widespread local transmission had confirmed or possible laboratory evidence of infection. In areas with limited local transmission, the prevalence increased 20% during October–December 2016, although not significantly; no increase was observed in areas without local transmission.

Compared with the previous report (*4*), this analysis added seven more jurisdictions (including one with widespread local transmission) and reported 18 months of data from monitoring births potentially affected by the outbreak. The previous report grouped widespread and limited local transmission areas together, reporting a 21% increase in prevalence for these areas combined (*4*). Stratification by local transmission levels provides support that the significant increase in prevalence is exclusive to widespread local Zika virus transmission areas. Further, the baseline prevalence of birth defects potentially related to Zika virus infection during the reference period in the 22 jurisdictions is consistent with the baseline prevalence for three jurisdictions before Zika virus was introduced in the Region of the Americas (*5*).

The findings in this report are subject to at least four limitations. First, results might not be generalizable beyond the included jurisdictions because jurisdictions might differ in population demographics and case-finding methodology. Second, heightened awareness can result in better identification of affected infants. For example, there might have been more extensive implementation of recommendations for eye exams in widespread local transmission areas. Third, categorization of areas with limited local transmission included regions of Florida and Texas that were larger than the actual areas of local transmission, which might mask any increase in Zika-related birth defects in smaller geographic areas where transmission occurred. Finally, the majority of cases did not have Zika virus testing reported. In widespread local transmission areas, approximately three quarters of cases had at least one sample tested, although the relatively high prevalence of negative results could reflect that timing might not have been optimal for detection of Zika virus in many cases. However, nearly half of those tested had laboratory evidence of Zika virus infection.

During the Zika virus outbreak, population-based birth defects surveillance programs were adapted to monitor birth defects potentially related to Zika virus infection during pregnancy. Use of population-based birth defects surveillance programs and the U.S. Zika Pregnancy and Infant Registry provide an example of a complementary approach in ascertaining both exposures and outcomes to better monitor new and emerging threats during pregnancy and impact on infants (*8*). Birth defects surveillance was important for identifying infants with birth defects potentially related to Zika virus infection whose mothers were not tested during pregnancy or were not tested at a time when infection could be detected. Health departments can use these data to inform referral services for affected infants and program planning. These findings underscore the important role of birth defects surveillance programs in preparing for emerging public health threats to pregnant women and infants.

**Summary**

**What is already known about this topic?**

In states and territories with documented local Zika virus transmission, the prevalence of birth defects potentially related to Zika virus infection during pregnancy increased 21% during the second half of 2016 compared with that in the first half.

**What is added by this report?**

In U.S. territories with widespread local Zika virus transmission, the prevalence of birth defects potentially related to Zika virus infection increased fourfold during January–March 2017 compared with January–March 2016.

**What are the implications for public health practice?**

During the Zika virus outbreak, birth defects surveillance programs adapted to rapidly identify Zika-related birth defects regardless of laboratory evidence. These data provide more complete information on all infants affected and allow planning for care.

## Footnotes

* With population-based surveillance of birth defects potentially related to Zika virus infection, information is collected on all infants who have birth defects that might be related to Zika virus infection. This includes infants who have not been exposed to Zika virus and might have the same birth defects for other reasons. https://www.cdc.gov/pregnancy/zika/research/birth-defects.html.

† 1) Brain abnormalities and/or microcephaly (congenital microcephaly [head circumference <3rd percentile for gestational age and sex, and documentation of microcephaly or a small head in the medical record], intracranial calcifications, cerebral atrophy, abnormal cortical gyral patterns [e.g., polymicrogyria, lissencephaly, pachygyria, schizencephaly, gray matter heterotopia], corpus callosum abnormalities, cerebellar abnormalities, porencephaly, hydranencephaly, ventriculomegaly/hydrocephaly [excluding “mild” ventriculomegaly without other brain abnormalities], fetal brain disruption sequence [collapsed skull, overlapping sutures, prominent occipital bone, scalp rugae], other major brain abnormalities); 2) neural tube defects and other early brain malformations (anencephaly/acrania, encephalocele, spina bifida, holoprosencephaly); 3) eye abnormalities without mention of a brain abnormality (microphthalmia/anophthalmia, coloboma, cataract, intraocular calcifications, chorioretinal anomalies [e.g., atrophy and scarring, gross pigmentary changes, excluding retinopathy of prematurity]; optic nerve atrophy, pallor, and other optic nerve abnormalities); 4) consequences of central nervous system dysfunction (arthrogryposis, clubfoot with associated brain abnormalities, congenital hip dysplasia with associated brain abnormalities, and congenital sensorineural hearing loss).

§ Overall and in each jurisdictional group, there were no changes in the prevalence of NTDs and other early brain malformations during January 2016–June 2017. There were 1,170 cases of NTDs and other early brain malformations and 1,547 cases of consequences of central nervous system dysfunction in this period.

¶ Areas with limited local transmission of Zika virus: southern Florida counties and Texas Public Health Region 11 (https://www.cdc.gov/media/releases/2017/s0313-risk-of-zika-transmission-florida.html, https://emergency.cdc.gov/han/han00393.asp, https://emergency.cdc.gov/han/han00399.asp). Areas without local transmission of Zika virus: California [selected counties], Georgia [selected metropolitan Atlanta counties], Hawaii, Illinois, Indiana, Iowa, Louisiana, Massachusetts, Minnesota, New Jersey, New York [excluding New York City residents], North Carolina [selected regions], Oklahoma, Rhode Island, South Carolina, Texas Public Health Region 10, Utah, Vermont, and Virginia.
